# Noise-free simulation of an FT-IR imaging hyperspectral dataset of pancreatic biopsy core bound by experiment

**DOI:** 10.1038/s41597-019-0260-x

**Published:** 2019-10-29

**Authors:** Tomasz P. Wrobel, Paulina Koziol, Magda K. Raczkowska, Danuta Liberda, Czeslawa Paluszkiewicz, Wojciech M. Kwiatek

**Affiliations:** 1 Institute of Nuclear Physics Polish Academy of Sciences, PL-31342 Krakow, Poland; 2 Faculty of Physics and Applied Computer Science, AGH University of Science and Technology, Mickiewicza 30, Krakow, Poland

**Keywords:** Imaging and sensing, Cancer imaging

## Abstract

A noise-free hyperspectral FT-IR imaging dataset of a pancreatic tissue core was simulated based on experimental data that allows to test the performance of various data analysis and processing algorithms. A set of experimental noise levels was also added and used for denoising approaches comparison, which due to the noise-free reference signal enables to truly observe signal distortion caused by different approaches.

## Abstract (summary)

**Table:** 

Measurement(s)	pancreas
Technology Type(s)	Fourier transform infrared spectroscopy • hyperspectral imaging
Factor Type(s)	nucleic acid • protein • lipid • wavelength • noise

Machine-accessible metadata file describing the reported data: 10.6084/m9.figshare.9959129

## Background & Summary

IR Imaging and spectroscopy is a fast developing field with new applications and branches of research emerging every year^1–3^. Some of the applications are well within the current capabilities of the technique, while others are still pushing the limit. High speed, High Definition imaging would be desirable for a clinical translation^4,5^, however, obtaining useful Signal to Noise Ratio (SNR) usually requires averaging multiple acquisitions. Pre-processing^6,7^ and denoising in particular plays a major role here. However, few approaches give full insight into their influence on signal, since any noise rejection may result in distorted data. Moreover, every experimental signal will have a noise component even after long acquisitions. In order to have a true insight into signal distortion we decided to create simulated datasets with raw and noisy data^8,9^.

To ensure the highest reliability of the simulations, experimental data was taken to provide spectral and spatial information used as the initial starting point. A tissue biopsy core was measured and cluster analysis was done to determine three main classes distribution. Afterwards, these spatial concentration profiles were assigned spectral data profiles of proteins, nucleic acids and lipids, which were based on spectroscopic knowledge and heavily randomized to ensure high variability as expected in a biological material. Still, only three profiles were used and a larger number of classes may be used to capture more of real biological material heterogeneity. Finally, the summed spectral profiles of varying spatial concentrations formed an initial dataset, which was later corrupted with randomized baseline. However, this dataset did not take into account the dependence of spatial resolution from the wavelength of light (the spectral frequency), since the concentration profiles obtained from cluster analysis removed it during clustering. In the next step such dependence was introduced and a full noise-free dataset was obtained. Afterwards, varying levels of noise, derived from experimental measurements, corresponding to increasing number of acquisitions were introduced to form different SNR datasets for denoising.

The motivation behind this research was to investigate available denoising approaches, not only by estimating improvement done by noise reduction but also by having insight into the degree of distortion done to a signal. This kind of analysis was never done before, especially on that scale. We recently published results, where among others we presented optimization process of five spectral denoising techniques^8^. By making this data available to other researchers we aim to encourage them to perform similar optimizations, which is possible not only for denoising but might be applied to diversity of preprocessing techniques. These datasets are a perfect platform to test and objectively compare state-of-the-art and new algorithms of any kind of data treatment with denoising being the intended use for this simulations.

## Methods

A typical FT-IR imaging dataset consists of a data cube, which spectral dimension is usually approximately 800 or 1600 variables depending on the spectral range and resolution, 8 or 4 cm^−1^, respectively. The spatial x and y dimension vary a lot – depending on the samples and magnification optics, dictating the projected pixel size – still a range from hundreds to dozens of thousands reported in the literature.

In our datasets, the complete process of obtaining a simulated FT-IR dataset consists of two main parts: experiment and simulation. The purpose of the experiment was to obtain an actual estimation of the data structure, which will form the basis of the simulation. The briefly outlined here process lead to creation of simulated FT-IR datasets directly based on experimental parameters.

1. Pancreatic biopsy tissue core was measured to create a full image, and thus obtain the spatial structure of the dataset.
2. Small subset (a 64 × 64 pixel area) was measured with varying number of acquisitions giving information about spectral noise levels.
3. Initial spectra were simulated with Voigt profiles and randomized.
4. Baseline was added to achieve more realistic (disturbed by artifacts) data.
5. Wavelength dependence of the spatial resolution was introduced using Gaussian filter imitating an Airy’s disc.
6. Various levels of noise were added creating range of simulated data with different SNR.

More detailed description is presented below.

### Experimental

The experimental part was based on a Tissue Micro Array (TMA) with pancreatic cancer tissue cores, purchased from US Biomax Inc., which also holds informed consents from human subjects for research. A paraffin embedded, 5 µm thick section was mounted on BaF_2_ salt window for transmission measurements mode. 24 h long hexane bath was performed for sample deparaffinization. The equipment used for FT-IR imaging was Bruker Vertex70v Spectrometer coupled to Hyperion 3000 microscope with Focal Plane Array (FPA) detector 64 × 64 pixels and 36x objective (projected pixel size of 1.1 µm).

#### Data spatial structure

**Fig. 1:**
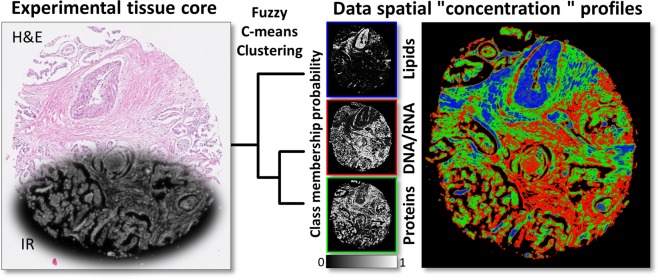
Graphical illustration of the process leading to data spatial structure characterization. FT-IR data (left) served as a base for Fuzzy C-means Clustering, which led to obtaining three spatial profiles (right) including proteins, nucleic acids and lipids. Black and white images with color frames present the class membership probability of each profile separately, and RGB image shows a combination of all.

A single pancreatic tissue core with a diameter of approximately 1 mm was measured in transmission mode, giving around 700 000 sample pixels and 600 000 background pixels. Data was acquired in the range from 3850 cm^−1^ to 900 cm^−1^ with spectral resolution of 8 cm^−1^ and 4 scans averaging. The normally used in the Fourier transform zero filling factor of 1 results in interpolated data and the final spectral spacing is equal to 4 cm^−1^. Data were written in a binary file with single precision giving a file size of 3.8 GB. Unsupervised Machine Learning in the form of simple Cluster Analysis is known to show good results in FT-IR Imaging^10,11^. Therefore, in order to prepare data for further analysis, Principal Component Analysis (PCA) denoising with 50 components was applied. After empty pixels exclusion, Fuzzy C-means Clustering (FCM) with D-Values as a distance measure (modified Pearson correlation coefficient)^10^ was performed using algorithm implemented in CytoSpec software. The number of classes was arbitrarily chosen as three, since the goal was to obtain spatial profiles of three main biochemical classes. The general scheme and outcome of this process are shown in Fig. 1. Two more spatially similar classes were assigned to protein and DNA/RNA classes, respectively, while the more distinct one to lipids, as is often the case in biological tissues. It later served as a base for spatial arrangement (concentration scaling factors) during simulation process. Additionally, the image for Amide I band (1650 cm^−1^) of experimental data was used for tissue mask creation. The applied threshold was equal to 0.15. Pixels with absorbance above this limit were marked with 0 (tissue region) and 1 if opposite (background region).

#### Noise levels estimation

**Fig. 2:**
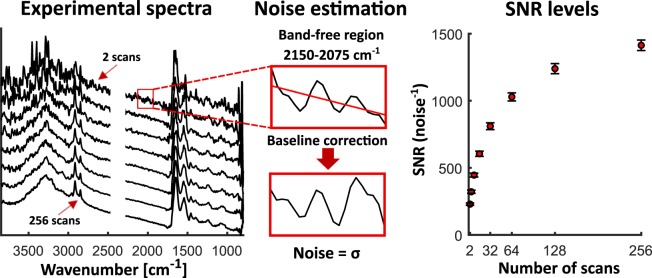
Procedure for experimental noise estimation. Examples of experimental spectra with different noise level corresponding to 2–256 scans averaging (left). (middle) Presentation of band free region, with a fitted straight line, used for noise estimation. After baseline correction, standard deviation gives numerical noise value (middle). SNR presented as an average of results for sample region pixels (spectra), for datasets corresponding to the number of scans presented on X axis (right).

Six areas of 64 × 64 pixels (corresponding to a single tile), partly covering pancreatic tissue and partly background area were measured in transmission mode. In this case, the spectral range was from 3850 cm^−1^ to 800 cm^−1^, with 8 cm^−1^ resolution. These measurements were repeated 8 times, for 2, 4, 8, 16, 32, 64, 128 and 256 scans averaging (and 64 for background measurement) and giving experimental spectra with different signal quality as presented in the left part of Fig. 2.

In order to numerically estimate noise levels corresponding to each dataset, a noise describing metric was implemented. Like it is shown in the middle part of Fig. 2, noise estimation consists of two steps. First, a straight line is fitted to the band free region of the spectrum (2150–2075 cm^−1^). Afterwards, a line is subtracted and the absolute value is taken:

$${x}_{i}=\left|{y}_{i}-{b}_{i}\right|,$$

where: *y*_*i*_ is a raw data point and *b*_*i*_ is a corresponding data point of the fitted line. Secondly, the results from the previous step served for the standard deviation calculations giving noise values for a given spectrum:

$$noise=\sqrt{\frac{{\sum }_{i}^{K}{\left({x}_{i}-\bar{x}\right)}^{2}}{N-1}},$$

where: $\bar{x}$ is a mean value of *x*_*i*_, *i* and *K* are the indexes of 2150–2075 cm^−1^ data range and *N* is a number of points. A MATLAB implementation of the presented above algorithm is provided with the code set. The obtained results of Signal to Noise Ratio (SNR) with a signal of unity (signal value taken as 1) are presented in Fig. 2. For better statistics and results stability the SNRs for pixels in the sample region were averaged for each dataset. We observed here a good SNR shape, which was expected to be proportional to the square root of number of scans. This result was later used for the estimation of the amount of noise added to simulated spectra.

### Simulations

**Fig. 3:**
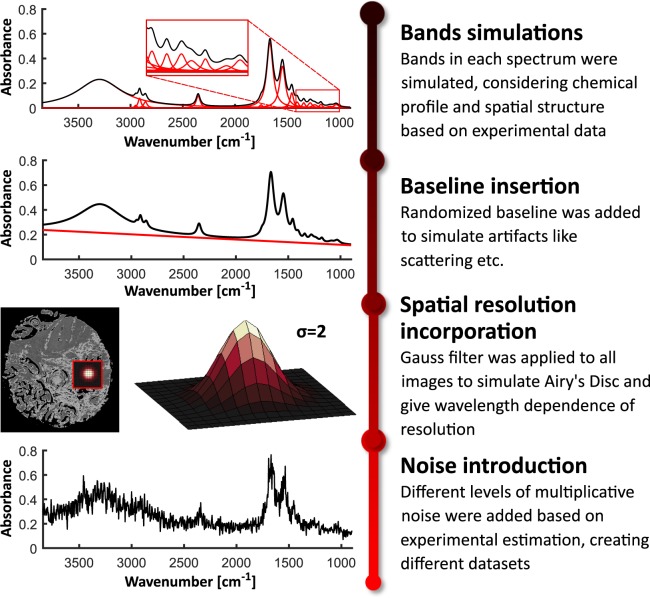
Scheme describing the workflow of the simulation process. First part included creation of spectra with determined chemical profile using Voigt profiles as bands approximation. Next step was aimed to insert randomized baseline in order to introduce artifacts to the system. Afterwards, spatial resolution dependence was introduced by using a Gauss filter. Finally, different levels of multiplicative noise were added to clean data.

The core part of this data descriptor is the process of simulation of a reliable dataset being a good imitation of FT-IR imaging data. The whole process consists of four main steps described below with a general workflow presented in Fig. 3. This part of analysis was entirely done in MATLAB Software.

#### Spectral profiles generation

**Fig. 4:**
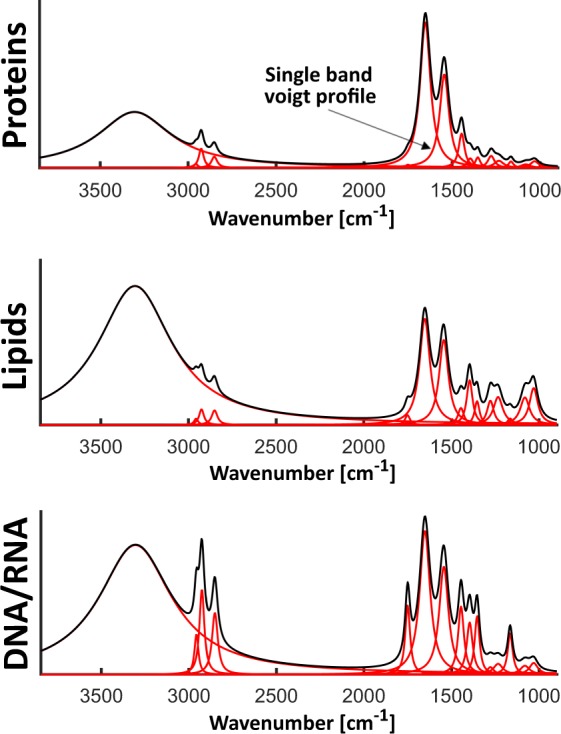
Simulated spectral profiles of the three main components: proteins, lipids and DNA/RNA.

In this part of the simulation, each spectrum of the hyperspectral dataset was simulated individually and later arranged in a spatial manner via spatial “concentration” profiles, which are linear combination coefficients of the three profiles. An important remark to make is the definition of single band shape. In general, IR bands are with a good approximation similar to Gaussian or Lorentzian-like curves^12^. However, more specific sources claim that closer estimation is given by a Voigt profile^13^, which is defined as a convolution of Gauss function and Lorentz function^14,15^. Here the Voigt profile implementation made by Sanjar M. Abrarov and Brendan M. Quine from York University in Canada was used. Individually simulated profiles contain 16 bands with defined position and width of Voigt functions (50% Gaussian and 50% Lorentz functions). These profiles are later multiplied by corresponding scaling factors obtained from cluster analysis of experimental data and summed giving the shape of final spectrum. In other words, it is a sum of three components corresponding to data profiles determined in the experimental section - proteins, nucleic acid and lipids. Spectral characteristics of the base profiles used in simulation are presented in Table 1. They were defined by an experienced spectroscopist to give typical shapes and ratios of bands of the main profiles – typical profiles are shown in Fig. 4. During the simulation process, all parameters were randomized with various levels of standard deviation (Table 1). Spectra were simulated in the range from around 3850 cm^−1^ to 900 cm^−1^ with 4 cm^−1^ resolution, although in the provided scripts bands positions and widths were converted from wavenumbers into points. After generation and randomization of the three profiles for each of the pixels, the three profiles were summed according to spatial concentration profiles. An exception was made for CO_2_ band (2352 cm^−1^), since it is a random component, not related in concentration to any spatial structure. Therefore, its concentration profile was completely random spatially, while not randomized in terms of width. In order to reach more realistic tissue section absorbance values whole the dataset was multiplied by the factor of 10. Some additional minor steps were also applied, but it can be concluded from provided scripts.

**Table 1:** Information/data used in spectral profiles simulations.

Band position [cm^−1^]	Randomization of position – in term of SDT	Band width [cm^−1^]	Randomization of width – in term of SDT	Voigt profile area - DNA/RNA	Voigt profile area - proteins	Voigt profile area- lipids
3300	1.8%	308	1%	4,000	3,000	1,800
2962	0.7%	15	1%	0,010	0,010	0,030
2920	0.6%	19	1%	0,030	0,060	0,080
2850	0.6%	23	1%	0,030	0,040	0,070
2352	0.4%	27	—	0,100	0,100	0,100
1754	0.3%	19	1%	0,020	0,010	0,070
1650	0.5%	42	1%	0,400	1,000	0,300
1550	0.4%	39	1%	0,300	0,600	0,200
1462	0.3%	23	1%	0,040	0,120	0,080
1400	0.2%	23	1%	0,100	0,037	0,060
1342	0.2%	19	1%	0,045	0,032	0,050
1280	0.2%	27	1%	0,060	0,050	0,010
1242	0.2%	39	1%	0,100	0,040	0,020
1172	0.2%	19	1%	0,012	0,020	0,040
1080	0.2%	39	1%	0,100	0,020	0,015
1030	0.2%	35	1%	0,120	0,035	0,020
				Randomization with SDT of 5%		

#### Baseline insertion

Experimental data are almost never artifact free and this fact must be included in the simulation. A group of artifacts distorting spectra with an appearance of a varying baseline is related to scattering effects and strongly depend on sample geometry and morphology. Scattering is approximately proportional to the wavenumber^16^ (light frequency) apart from situation where measured object is spherical with diameter close to the wavelength (for example cells)^17–19^. This is not the case for tissue measurements, therefore, a baseline in the form of a straight line was introduced here. Both line coefficients were randomized, and once again based on the spectroscopic knowledge, slope range was set from 0.15 to 0.25 and y-intercept was in the range from −0.00026 to 0. Baseline added to background spectra was divided by the factor of 15. This comes from the fact that the lack of sample greatly reduces scattering effects. An exemplary baseline is presented in the proper section of Fig. 3.

#### Spatial resolution dependence on wavelength

Since the simulated hyperspectral data should be representative of IR imaging technique, it was essential to take into account image formation physics related to the dependence of spatial resolution from the wavelength of light at the diffraction limit. Light spot coming from circular aperture is described by Airy’s disc^16^, with radius limited by the first minimum and described by:

$$r=1.22\cdot \frac{\lambda \cdot R}{a},$$

where *λ* is wavelength, *a* is aperture diameter and $R=\frac{a}{2.44\cdot NA}$ is a distance of focus from aperture described by numerical aperture *NA*. This leads to a more commonly known expression of:

$$r=\frac{\lambda }{2\cdot NA}.$$

With a typical NA of FT-IR imaging in the 0.5 range this leads to almost direct proportionality of the wavelength of light and the radius of an Airy’s disk. A reasonable approximation of the disk can be done with a Normal Distribution, therefore a Gauss spatial filter was applied to all images with individually computed filter parameter:

$$\sigma =34.1{\rm{ \% }}\cdot r.$$

Estimation of *σ* was done, by assuming that *r* is covered by three standard deviations of Gaussian distribution (3*σ*). This means that for a wavelength of 6 µm (amide I band at 1650 cm^−1^) the total distribution should cover 12 µm, which means it is covering almost 11 pixels in x and 11 in y (projected pixel size of 1.1 µm was used to obtain the spatial structure). In this step the data gained another important source of variability coming from the dependence of images (structures) on wavelength. Gauss filtering was the final step in the creation of raw FT-IR dataset simulating clean (noise free) signal.

#### Noise insertion

The main purpose of this simulation was to obtain datasets with different levels of noise and strongly bound by experimental data. Based on knowledge about the multiplicative character of noise in FT-IR spectroscopy^20^, noise level for maximal absorbance in the dataset was set to be 40% higher than for the minimal absorbance value. To execute this condition a Gaussian noise was simulated at first. Noise values were later linearly scaled with coefficients found from a system of two linear equations obtained for maximal and minimal value of absorbance:

$$a=nois{e}_{G}\cdot \frac{MIN-1.4\cdot MAX}{MIN-MAX},$$

$$b=nois{e}_{G}\cdot a\cdot MAX,$$

where *a* is slope for noise scaling, *b* is y-intercept, *noise*_*G*_ is Gaussian noise and *MIN* and *MAX* are minimal and maximal values in whole raw dataset. Final noise added to the system was:

$$nois{e}_{M}=a\cdot {A}_{ijk}+b,$$

where *A*_*ijk*_ is absorbance for a given data point of hyperspectral data. The parameter that allowed to manipulate the final level of noise was the standard deviation of initially generated Gaussian noise. To reach the desired noise level (corresponding to experimental dataset) an iterative process was conducted. The standard deviation of Gaussian distribution used in noise generation was set to the value of noise for the experimental dataset. After introduction of generated noise into raw data, noise estimation was performed with described earlier SNR metric. If the obtained value differed from the experimental one, process was repeated with proper correction in standard deviation of Gauss noise. Due to the size of raw dataset (around 4 GB) this process might be time consuming and it is recommended to conduct it on a computer with sufficient RAM memory. The approach described here allows simulation of any noise level. 8 datasets with signal quality corresponding to 2, 4, 8, 16, 32, 64, 128 and 256 scans averaging were created.

## Data Records

Simulated datasets and above-mentioned scripts and algorithms are available at the Open Science Framework^9^.

Main data including raw and noisy datasets were stored in Matlab.mat format. Each file is around 3.2 GB and after loading into workspace has three dimensions. First and second corresponds to a spatial position (pixels), third dimension refers to spectral variables. For example, extracted image of any spectral variable gives matrix with size of 1180 × 1100, but extracted single spectrum gives vector of size 1 × 1 × 765 and needs reshape (or squeeze) function to be plotted.

Besides final datasets we also provided results of experiment, which are directly needed for the simulations. Results of cluster analysis giving scaling factors for each data profile are available in .mat format. This file contains four variables, three variables with size of 1180 × 1100 corresponding nucleic acids (firs class), proteins (second class) and lipids (third class). Last variable is a combination of all three with size of 1180 × 1100 × 3 and the same order (might be used for RGB image). Results of noise estimating experiment are provided in excel file with proper description inside.

Data mask determining sample and background regions is in the form of .mat file with logical data type where 0 corresponds to sample pixel and 1 to background pixel.

Wavelength vector (.mat file) with dimensions of 765 × 1 is provided for the potential user in case if determining spectral regions is needed.

Scripts and functions have Matlab .m format, but might be open with any text editor.

## Technical Validation

### Principal component analysis

**Fig. 5:**
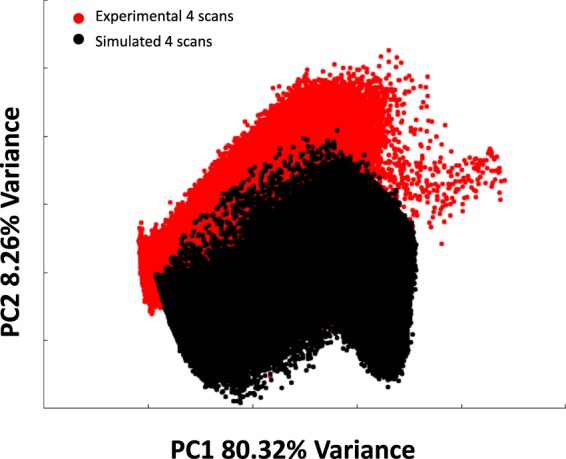
PC1 (80.32% variance) vs. PC2 (8.26% variance) scatter plot of the simulated dataset with 4 scans and of the experimental dataset with 4 scans.

The main potential limitation of trying to simulate spectra of biological origin is to have too little variability, as there are millions of different molecules contributing to the acquired signal. PCA is a convenient way of comparing variance sources^21,22^ in two datasets and was therefore performed on the original experimental dataset and a simulated one with noise levels of the same order (the same number of scans). The scatter plot of PC1 vs. PC2 is shown in Fig. 5.

It can be seen that there is a large overlap between the two datasets, especially on PC1, which accounts for 80% of variance. There is some shift in PC2 as was expected, since simulation will not capture all of the variability. The values of the simulated dataset were scaled by 1.6 to have a total max absorption of 1.6 at amide I position as was found in the experimental dataset.

## Usage Notes

A huge advantage of the provided here data is a presence of clean (noise free) dataset. Since preprocessing applied to more realistic noisy data might affect them in an unexpected way, therefore it is useful to compare introduced changes with the initial signal. This was exactly the case in the study investigating the influence of different denoising techniques on FT-IR data^8^. To numerically estimate level of deformation done by denoising, a Signal Distortion (SD) metrics was defined and it consists of the following steps. Initial step was to find an absolute value of noise introduced to the system, as:

$$nois{e}_{ijkabs}=\left|{A}_{ijknoisy}-{A}_{ijkclean}\right|,$$

where *A*_*ijk noisy*_ is absorbance for data point of noisy dataset and *A*_*ijk clean*_ is a corresponding data point from raw dataset. Next, an absolute value of noise left after denoising was estimated:

$$nois{e}_{ijkabsred}=\left|{A}_{ijkdenoised}-{A}_{ijknoisy}\right|,$$

where *A*_*ijk denoised*_ is absorbance for data point of denoised dataset. Final part was to sum values of:

$$S{D}_{ijk}=\left|nois{e}_{ijkabsred}-nois{e}_{ijkabs}\right|,$$

but only for *ijk* data points where *noise*_*ijk abs red*_ > *noise*_*ijk abs*_. This additional condition was introduced to avoid situation where the distortions caused by denoising were smaller than the original noise. This custom made metrics was created for the purpose of denoising but it might be easily adapted for other applications and we recommend to use whenever reference signal is needed. MATLAB script for SD computation is also provided.

## Data Availability

Simulated datasets and mentioned above scripts and algorithms are available at the Open Science Framework^9^. MATLAB Software version R2017b was used for the creation of scripts with additional toolboxes: Statistics and Machine Learning and Image Processing. CystoSpec Software version 2.00.01 was used for PCA denoising and FCM clustering. Voigt profile computation algorithm implemented by Sanjar M. Abrarov and Brendan M. Quine is available at: https://ww2.mathworks.cn/matlabcentral/fileexchange/47801-the-voigt-complex-error-function-second-version.
